# What is *Xenoturbella*?

**DOI:** 10.1186/s40851-015-0018-z

**Published:** 2015-07-24

**Authors:** Hiroaki Nakano

**Affiliations:** Shimoda Marine Research Center, University of Tsukuba, 5-10-1, Shimoda, Shizuoka, 415-0025 Japan

**Keywords:** *Xenoturbella*, Acoelomorpha, Xenacoelomorpha, Deuterostome, Bilateria, Metazoa, Larva, Development, Phylogeny, Evolution

## Abstract

*Xenoturbella* is a strange marine worm that can be collected regularly only off the west coast of Sweden. Due to its simple morphology, which lacks a centralized nervous system, coelom, anus, or reproductive organs, its phylogenetic position has long remained obscure. Recent phylogenomic analyses suggest it forms a new phylum, Xenacoelomorpha, together with the Acoelomorpha, but the position of the phylum remains undecided, either as a deuterostome or an early branching bilaterian. Developmental stages exhibit many phylogenetically decisive characters in various animal species, but have remained a mystery for *Xenoturbella* until recently. Observations of its development showed it has direct development with a very short and simple swimming stage, and that it lacks a feeding larva. Asexual reproduction has never been reported. It has been suggested that *Xenoturbella* feeds specifically on bivalves, but it still remains unknown whether it feeds on sperm, eggs, larvae, juveniles, carcass, mucus, or feces of bivalves, and direct observations of *Xenoturbella* feeding on bivalves have not been reported. Endosymbiont bacteria have been found, and their functions are being investigated. The evolutionary scenario of this taxon remains the subject of debate, and our understanding will depend largely on determining its phylogeny. Thus, although recent studies have uncovered many new and crucial facts regarding *Xenoturbella*, some fundamental biological information, such as phylogeny, complete life cycle, and genome, remain unsolved. Further research on the well-studied Swedish *Xenoturbella bocki*, as well as the discovery of new species elsewhere, are necessary if we are to more fully understand the nature of *Xenoturbella*.

## Introduction

“Was ist *Xenoturbella*?”

Reisinger, E. *Z. Wiss. Zool.*: 164, 188–198 (1960) [1]

**Fig. 1:**
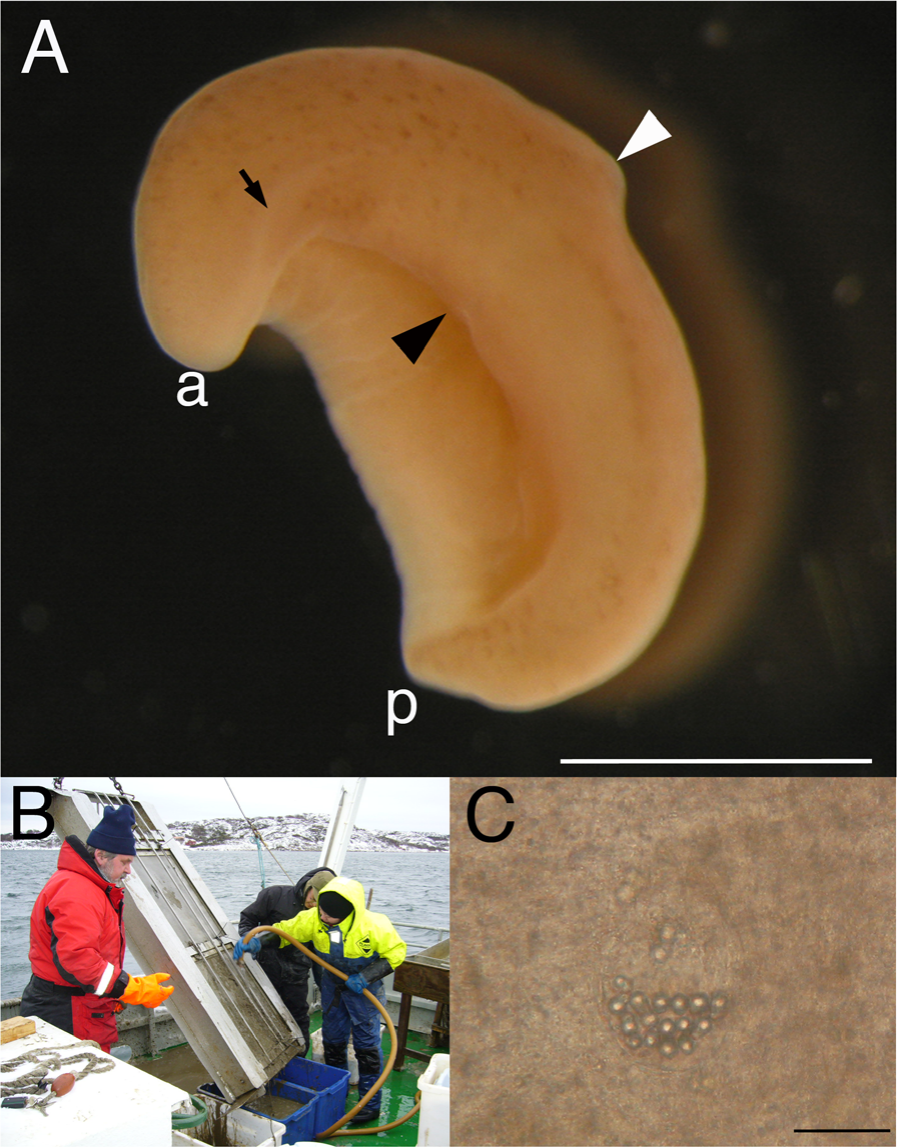
*Xenoturbella* morphology and collections. **a** External morphology of *Xenoturbella bocki*. Side furrows (black arrow) are present on the lateral sides from the anterior tip (a), but these do not reach the posterior end (p). The mouth (black arrowhead) is situated on the ventral side anterior to the circumferential furrow (white arrowhead). Scale bar: 1 cm. **b** *Xenoturbella* is collected at Gullmarsfjord, Sweden, using a Warén’s dredge and the research vessel Oscar von Sydow, Sven Lovén Centre- Kristineberg, Gothenburg University. Mud is brought up from the sea bottom with the dredge, and is sieved to search for the animal. **c** Statocyst of an animal pressed between cover and slide glasses. Round cells are observed within the organ. Scale bar: 50 μm

*Xenoturbella* is an enigmatic marine worm with a very simple morphology (Fig. 1a). Due to its simplicity, the animal’s phylogenetic position has remained obscure since its first scientific report in 1949 [2]. In 1960, Dr. Reisinger even published a manuscript entitled ‘Was ist *Xenoturbella*?’ [1] (English translation: What is *Xenoturbella*?).

After recent molecular phylogenetic and phylogenomic analyses have shown that it may occupy an important phylogenetic position with respect to the evolution of deuterostomes and metazoans [3–5], *Xenoturbella* has attracted the interest of many biologists. However, since the animal can be collected regularly only off the west coast of Sweden (Fig. 1b) [6], studies of its development and ecology have been lagging. In this review, I will summarize the recent studies on *Xenoturbella*, and examine how close we are to answering Dr. Reisinger’s pointed question.

## Review

### Morphology

*Xenoturbella* is a marine worm about 1–3 cm long possessing a very simple body plan [2]. Externally, it has a brownish to pale yellow color with a whitish anterior (Fig. 1a). Black spots can be seen on many of the specimens. The circumferential furrow encircles the body approximately at the middle, and side furrows are seen running down both lateral sides from the anterior tip to around the circumferential furrow. The mouth opens on the ventral side, slightly anterior to the circumferential furrow. Concerning internal morphology [2], the outermost layer is the epidermis with cilia used for locomotion. There is an intraepidermal nerve net [7–9], which is slightly thickened beneath the side furrows and around the statocyst (see below). Interior to the basal lamina is the muscle layer, with the circular muscles on the outside and the longitudinal muscles inside. The innermost structure is the intestine, which is connected to the outside via the mouth, but an anus is absent, making the digestive organ sac-shaped, rather than the tube-shape generally seen in bilaterian animals. There is an organ, called the ‘statocyst,’ near the anterior tip (Fig. 1c), but its morphology differs greatly from the statocyst seen in other marine invertebrates. It is thus unclear whether it actually exhibits a balance-sensing function; endocrinal functions have also been suggested [10]. Similarly, the side furrows have been suggested as a sensory organ [2], but their function has not been demonstrated yet. Structures such as a central nervous system (brains or ganglia), reproductive organs (gonads or gonopores), excretory organs, or coeloms, generally seen in bilaterians, are lacking in *Xenoturbella*.

### Phylogeny

**Fig. 2:**
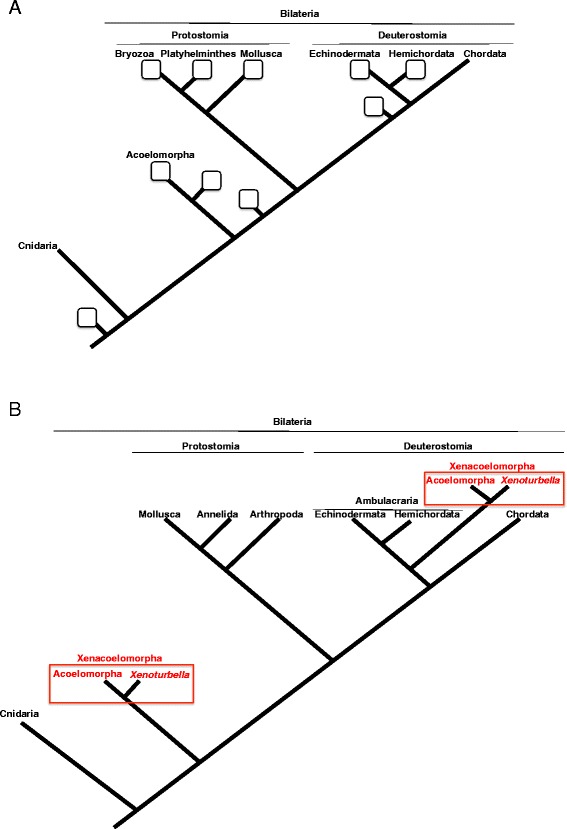
Phylogenetic position of *Xenoturbella*. **a** Squares indicate phylogenetic positions previously suggested for *Xenoturbella*, based mainly on morphology. **b** Two competing theories for the phylogenetic position of *Xenoturbella* based on recent phylogenomic analyses. Both hypotheses suggest a close relationship with the Acoelomorpha, forming the phylum Xenacoelomorpha

*Xenoturbella* could be considered the champion wanderer of the phylogenetic tree (Fig. 2a). Original description in 1949 regarded the animal as a peculiar primitive turbellarian within the phylum Platyhelminthes [2]. But over the next 60 years, relationships to many other animal groups have been suggested. Jagersten suggested it was a plesiomorphic metazoan group based on the simple body plan [11]. A hypothesis suggesting that it is a basal bilaterian has been proposed as well [7, 12]. A basal deuterostome affinity was suggested based on similarities in the epidermal structure [1, 13]. Its statocyst was reported as being similar to those of echinoderm sea cucumbers [1], whereas its myocytes were suggested to be similar to those of the hemichordates [1]. Based mainly on morphological cladistics, a bryozoan affinity has been suggested [14]. *Xenoturbella* oocytes have been reported to be similar to those of bivalves [15]. The overall body plan and the epidermal cilia morphology suggested *Xenoturbella* was either related to, or a member of, Acoelomorpha [2, 16–19]. Acoelomorpha is another group of enigmatic marine worms with a simple morphology, which was first suggested as belonging to the phylum Platyhelminthes, but was subsequently moved to its own phylum at the base of the bilaterians, based on molecular phylogenetics [20]. To summarize, since the *Xenoturbella* adult morphology is so simple, with many organs absent, and since its larval morphology has been unavailable until recently (see below), it has been impossible to determine *Xenoturbella*’s position in the phylogenetic tree.

### Molecular phylogeny and phylogenomics

Molecular phylogenetics showed promise to solve the problem, but it added further confusion. The first application of using DNA sequences to determine the phylogeny of *Xenoturbella* resulted in the animal being grouped within the bivalves in the phylum Mollusca [21]. This result was surprising, as the animal possess little resemblance morphologically to bivalves, lacking any vestiges of shells or shell glands. In 2003, a second study, using the same genes 18S rRNA and COI, reported that *Xenoturbella* is a deuterostome [22]. Why then did these two studies, which used the same genes, yield different results? A simple experiment reported in the second study may answer this question. If DNA is extracted only from the epidermis of *Xenoturbella* and molecular phylogeny is performed, the results show the animal is a deuterostome. If DNA extracted from the gastrodermis is used, the animal is grouped within the bivalves. This suggests that the DNA used in the first report originated from bivalves that the animal preyed upon. Furthermore, subsequent molecular phylogenetic analyses using other genes supported the deuterostome relationship of the animal [3, 23–26]. Moreover, *Xenoturbella* has been shown to react positively to the Ambulacraria (echinoderms and hemichordates) specific SALMFamide-2 antibody [27]. Given these findings, the mystery was widely thought to have been solved, and it was generally accepted that *Xenoturbella* is a deuterostome.

However, in 2009, a phylogenomic study covering 94 taxa across the animal kingdom suggested that *Xenoturbella* is not a deuterostome [4], but rather a sister group to the Acoelomorpha, and that this group (*Xenoturbella* + Acoelomorpha) forms a sister clade to all other bilaterians (Fig. 2b).

Is *Xenoturbella* a deuterostome, or is it an early branching bilaterian related to the Acoelomorpha? The latest phylogenomic study supports a somewhat compromising hypothesis [5]. This 2011 study adopted mitochondrial protein sequences from *Xenoturbella* and Acoelomorpha to construct a phylogenetic tree. Independent analysis was also performed from a phylogenomic data set of 38,330 amino-acid positions. These two independent analyses both showed that the basal position of Acoelomorpha within the bilaterians is probably due to long-branch attraction, and that *Xenoturbella* and Acoelomorpha formed a clade within the deuterostomes related to Ambulacraria. Thirdly, the miRNA complements in *Xenoturbella* and Acoelomorpha were investigated, and deuterostome specific miRNAs were found from both groups. From these three independent data sets, the authors concluded that *Xenoturbella* and Acoelomorpha form a new phylum, ‘Xenacoelomorpha’, and that the phylum is a sister clade to the Ambulacraria within the deuterostomes (Fig. 2b).

There has been no major phylogenetic research focusing on *Xenoturbella* since. So, is the problem solved, is *Xenoturbella* a deuterostome? Biologists working on *Xenoturbella* all agree that it is not over yet, and that more studies, including phylogenetic analysis of morphological characters [28], are essential.

### Reproduction and development

Although the details of *Xenoturbella* gametogenesis or annual cycle remain unknown, it was reported as far back as the original 1949 description that winter is the most likely breeding season for the Gullmarsfjord population [2]. This was confirmed by recent studies [29], but what triggers spawning on the cold, dark sea floor at depths of about 100 meters has not been determined. One possible candidate is the annual inflow of deep water that occurs during winter in the Gullmarsfjord [30, 31], but more research is needed to definitively answer this question.

**Fig. 3:**
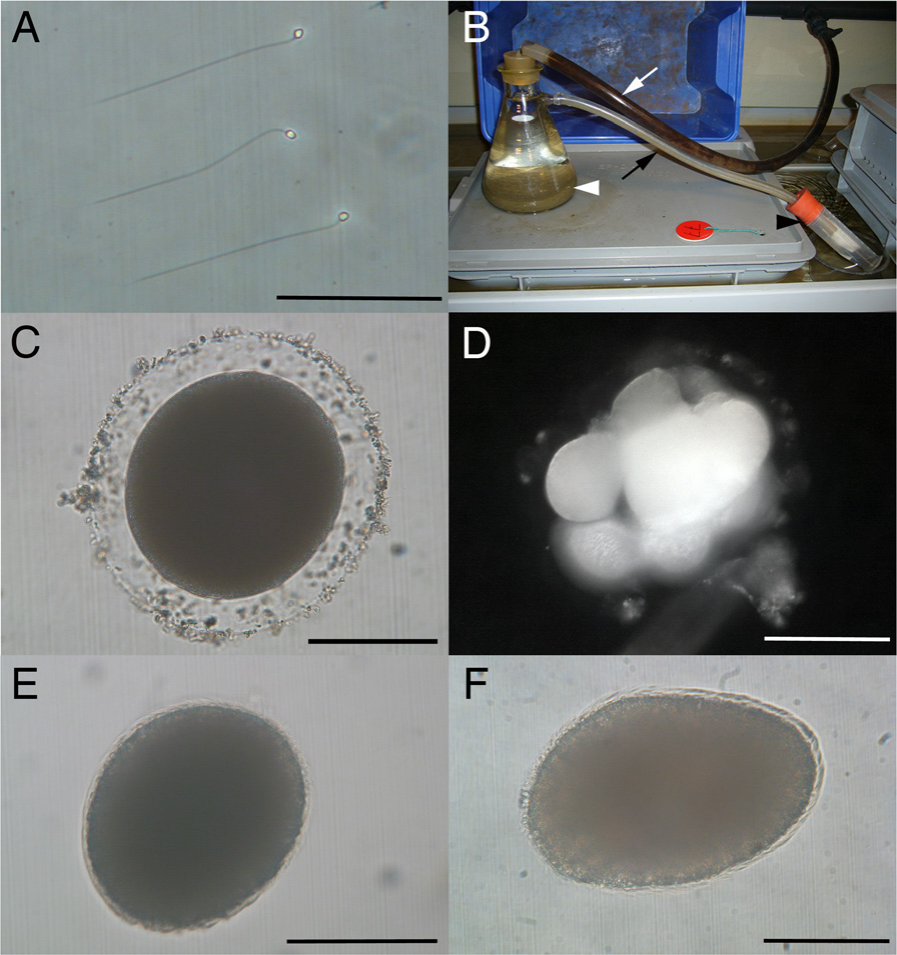
Reproduction and development of *Xenoturbella*. **a** *Xenoturbella* sperm dissected from an adult specimen. Scale bar: 10 μm. **b** Setup for collecting *Xenoturbella* eggs and embryos. Seawater enters the flask (white arrowhead) containing mud and *Xenoturbella* from the black hose (white arrow). Outflow from the flask passes through another hose (black arrow) and enters a tube with a mesh placed at the end (black arrowhead). This is just an example of many different experimental conditions that have been used, such as different containers, container size, presence/absence of running seawater, presence/absence of mud, and mesh size. **c** Egg found inside the tube with the mesh in the setup shown in (**b**). Scale bar: 100 μm. **d** Cleavage stage embryo, probably at the eight-cell stage. Scale bar: 100 μm. **e** Swimming stage uniformly ciliated embryo just after hatching, with the anterior to the top right. Scale bar: 100 μm. **f** Five days after hatching, with the anterior to the left. Scale bar: 100 μm

Sperm and eggs were described in 1949 as present in various sites within the adult body [2], and I have confirmed they are indeed present between the epidermis and intestine, attached to the outside of the intestine, between intestinal cells, and within the gastric cavity. Recent observations using electron microscopy have revealed that *Xenoturbella* sperm is a bilaterian primitive type, consisting of a round head without a separate midpiece, and a 9 + 2 single flagellum [32]. This type of sperm is found from a wide range of metazoans, mostly in species with external fertilization (Fig. 3a).

*Xenoturbella* developmental stages have long remained a mystery. A bivalve trochophore-like larva was found from adult section slides stored in the Swedish Natural History Museum and reported as a brooded *Xenoturbella* larva in 1999 [33]. However, it is now clear that *Xenoturbella* is not a mollusc, and that it probably feeds on bivalves [6, 22, 34, 35]. Furthermore, many researchers, myself included, have collectively made sections of over 100 adult specimens, and have failed to find a second brooded trochophore-like larva. Therefore, the trochophore-like larva is now widely regarded to be a bivalve larva consumed by the specimen, preserved in a largely undigested state in the gastrodermis at fixation.

It is possible to dissect out the eggs and sperm from mature *Xenoturbella*, but the gametes obtained in this way do not fertilize similar to some other marine invertebrates. Efforts to obtain viable gametes through spawning induction by physical or chemical stimulation have been unsuccessful, making it necessary to wait for natural spawning to occur. *Xenoturbella* was collected during the breeding season (December to March), and four to 20 specimens were kept together in containers in the laboratory [29]. The containers were checked for gametes and embryos several times a day. Some containers had running natural seawater, with a mesh placed on both the inflow and the outflow tubes (Fig. 3b). Others held still seawater, changed daily. Mud was sometimes placed in the containers. Eggs and embryos were discovered on several occasions (Fig. 3c), and DNA sequencing confirmed that these indeed belong to *Xenoturbella* [29]. On one occasion, cleavage stage embryos were found undergoing holoblastic radial cleavage on the mesh of the outflow tube (Fig. 3d), but development arrested after their removal from the containers, which was probably due to the damage of being pressed into the mesh by the water flow, as the embryos had a distorted shape. Hence, it still remains to be confirmed if this type of cleavage is normal for *Xenoturbella*.

On two occasions, swimming stage embryos were discovered from the containers, and an unhatched embryo was found on one of the occasions [29]. The yellowish embryos were uniformly ciliated, with an apical tuft at the anterior end (Fig. 3e). They lacked mouth, anus, vestibule, larval arms, ridges, and ciliary bands. The epidermis of the hatchlings is differentiated to a similar degree as in the adults, and the developing muscles and the nerve net can be seen internally [29]. No coelomata or gut were found in the embryos [29]. Five days after hatching (Fig. 3f), they started to glide on the bottom using their cilia and were able to contract using internal musculature, both behaviors similar to those observed in adults [29]. Therefore, *Xenoturbella* was discovered to undergo direct development, with a very short swimming stage, and to lack a feeding larva. The egg/embryo collection method described above (Fig. 3b) was instrumental in finally uncovering the developmental stages of *Xenoturbella* [29] since the first report of this animal in 1949 [2]. But some flaws of the method have been exposed in the process. First, the spawning time cannot be controlled, and this has led to missed opportunities to observe some important developmental stages, such as gastrulation. Second, water flow probably damaged the cleavage stage embryos. Last, it was impossible to obtain numerous embryos necessary for experiments such as gene expression analyses. Further research is needed to refine the egg/embryo collection method and to establish a developmental experimental system for *Xenoturbella*.

**Fig. 4:**
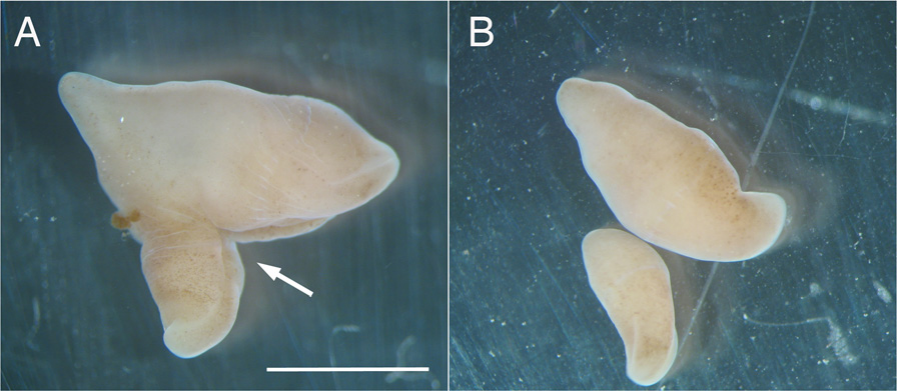
Division observed in *Xenoturbella*. **a** Individual with a wound (arrow) near the anterior end. Scale bar: 1 cm. **b** Same specimen as in (**a**), 12 days later. It has divided into two from the wound. The larger piece showed irregular behavior, and died five days later. The smaller piece exhibited normal behavior and survived

It is unclear whether *Xenoturbella* is able to reproduce asexually by fission or budding. I have studied the animal for about six years in Sweden, keeping specimens alive in the laboratory for most of the time, but have never observed fission or budding. I did observe instances in which damaged specimens divided into two or more pieces from a wound probably received during collection (Fig. 4). In these cases, the plane of division can be in any direction (longitudinal, latitudinal, diagonal, etc.), and some degree of tissue regeneration was seen in all pieces. But normal behavior and long-term survival was only seen in the piece with the statocyst, and although other pieces were able to glide using their cilia, they showed irregular behavior and did not survive long. Hence, it is likely that the statocyst is essential for survival and normal behavior in *Xenoturbella*, and the animal’s competence of asexual reproduction depends on the ability of new statocyst generation in adult specimens. I have performed several incision experiments, but was unable to confirm any statocyst generation. However, the possibility remains that environmental factors were not favorable in the laboratory aquarium. To summarize, asexual reproduction in *Xenoturbella* has not been observed and appears unlikely, but cannot be completely ruled out.

### Ecology

Little is known on *Xenoturbella* ecology. Specimens can be collected from muddy sea bottoms of about 50–150 m depths at the Gullmarsfjord on the west coast of Sweden, using the research vessel and Warén’s dredge of the Sven Lovén Centre- Kristineberg, Gothenburg University (Fig. 1b). Since the dredge scrapes off the mud from the surface layer of the sea bottom, it is assumed that *Xenoturbella* lives either on the surface of the mud or does not burrow deeply. However, animals in laboratory aquarium have been observed to dig tunnels deeper than 15 cm [36], and it is possible that they live deeper below the sea bottom in the wild. *Xenoturbella* glides using its cilia while excreting mucus around the body, but cannot swim. It can roll up into a ball using internal musculature, and can stay in that form for several months.

**Fig. 5:**
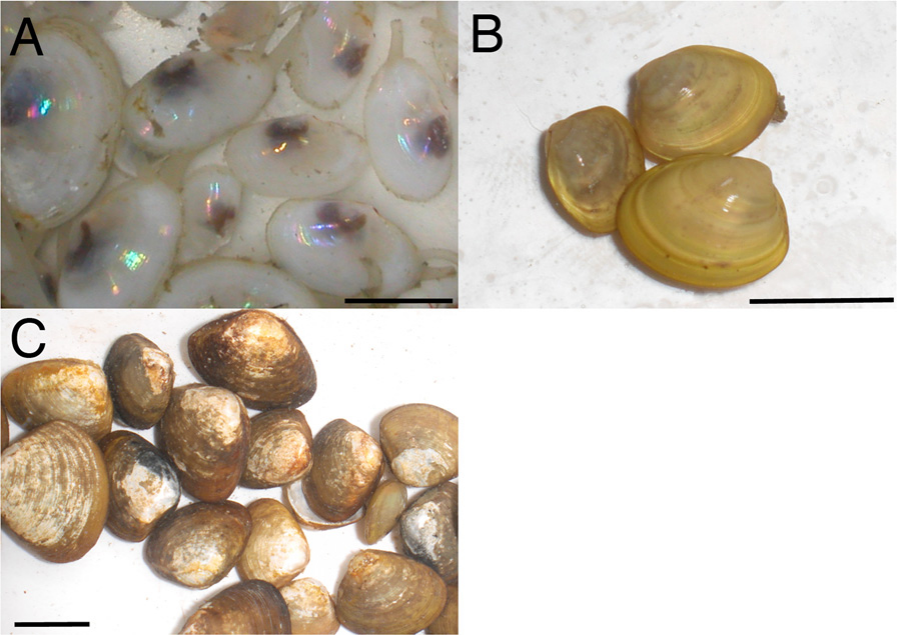
Bivalve species whose DNA have been identified from *Xenoturbella*. **a** *Abra nitida/alba*. **b** *Ennucula tenuis*. **c** *Nucula sulcata*. Scale bars: 1 cm

Only circumstantial evidence has been reported for the diet of *Xenoturbella*. A trophic level study using nitrogen isotopic compositions showed the animal has a high trophic level, and suggested that it feeds on other animals [37]. It is well documented that molluscan DNA contaminates the animal, and DNA from several species of bivalves that inhabit the same area as *Xenoturbella* have been identified from the animal (Fig. 5) [22, 34, 35]. Although annelids, arthropods, echinoderms, and other animals can also be collected together with *Xenoturbella*, only bivalve DNA is found from the animal, suggesting that *Xenoturbella* preys exlcusively on bivalves. And, since *Xenoturbella* apparently lacks any organs that could be used to pry open closed bivalve shells, it is reasonable to assume that they feed on small developmental stages of bivalves (sperm, eggs, larvae, and juveniles), dead bivalves, bivalve mucus, or bivalve feces. A bivalve trochophore-like larva has been reported within adult *Xenoturbella*, supporting this view [33]. With hopes of obtaining data on the *Xenoturbella* diet, we have performed preliminary experiments using bivalve sperm, eggs, larvae, juveniles, mucus, carcass, and feces (personal observations and [36]). Despite all the circumstantial evidence, *Xenoturbella*, even specimens starved for several months, were not attracted to any of the supposed food. One possibility is that the animal does not feed very often, since it can survive in seawater without any food, mud, or water change for over one year. It has also been suggested that the animal can uptake dissolved organic matter through its epidermis [38]. Long-scale behavioral research may be necessary to uncover the feeding nature of the animal.

Two types of endosymbiotic bacteria have been identified from *Xenoturbella*. Both the Chlamydia-like bacteria [39] and the *Gammaproteobacteria* “*Candidatus* Endoxenoturbella lovénii” [40] were abundant in the gut of the animal. They were also found in spermatid clusters of the animal, implying vertical symbiont transmission [40]. Although it is tempting to suggest that the bacteria played a role in loss of certain organs in *Xenoturbella*, or that the bacteria enables prolonged fasting in the animal, at present these are no more than speculative hypotheses, and much research is needed to uncover the role of these endosymbionts.

### Evolution

**Fig. 6:**
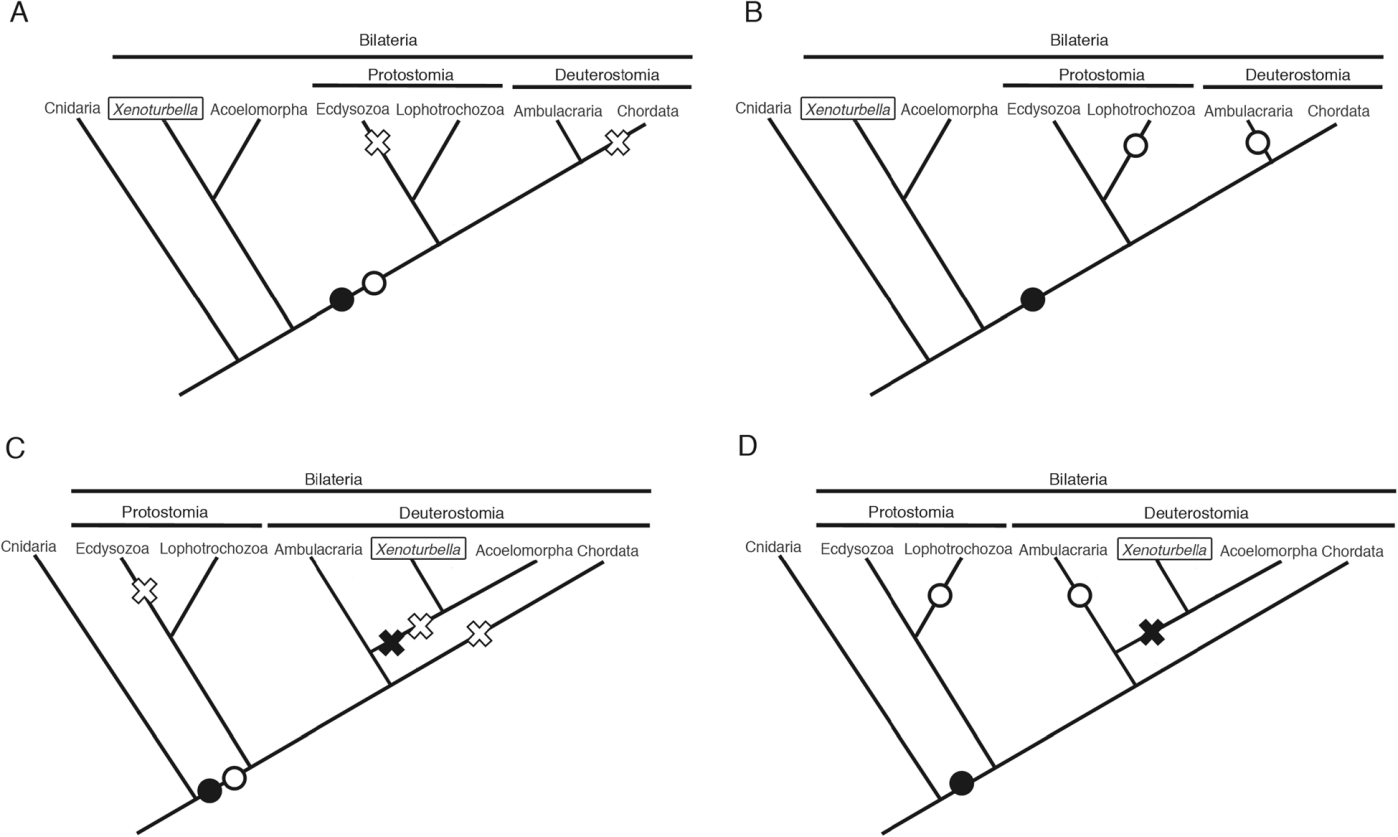
Different scenarios for the evolution of morphology and larva of *Xenoturbella*. **a, b** Phylogenetic trees showing Xenacoelomorpha as an early-branching bilaterian. **c, d** Phylogenetic trees showing Xenacoelomorpha as a deuterostome. Black circles show gain and black crosses show loss of adult organs (e.g. coeloms and anus). White circles show gain and white crosses show loss of feeding planktotrophic larva

The evolutionary history of *Xenoturbella* largely depends on the phylogenetic position of the animal. First, if it is an early branching bilaterian, it is likely that the simple body plan without a centralized nervous system and an anus was retained from the last common ancestor of bilaterians (Fig. 6a,b). Furthermore, the non-feeding uniformly ciliated larva found in Porifera, Cnidaria, and *Xenoturbella* probably represents the symplesiomorphy of metazoans.

In the second scenario, *Xenoturbella* and Acoelomorpha form a new phylum, ‘Xenacoelomorpha’, as a sister clade to the Ambulacraria within the deuterostomes (Fig. 6c,d). This theory implies that Xenacoelomorpha has secondarily lost several adult organs, such as anus, metanephridia, coeloms, and gill slits, all of which are believed to have been present in the deuterostome ancestor. As *Xenoturbella* are not sessile, parasitic, or truly microscopic, the cause for this secondary simplification remains to be uncovered. The situation on larval morphology is more complicated, depending on when the feeding larva, present in extant Ambulacraria and Lophotrochozoa, was acquired. If it was already present in the bilaterian ancestor, Xenacoelomorpha, Chordata, and Ecdysozoa have independently lost the feeding larva during evolution (Fig. 6c). This hypothesis will gain support if vestiges of digestive organs or coeloms are found from further research on *Xenoturbella* larva. On the other hand, if a feeding larval stage was acquired independently in Ambulacraria and Lophotrochozoa, it is likely that *Xenoturbella* has retained the symplesiomorphic larva of metazoans (Fig. 6d).

## Conclusions

So then, can we now answer the question, “What is *Xenoturbella*?” Even in 1960, when the paper with this as a title was published [1], *Xenoturbella* was known to be a marine invertebrate worm with a very simple body plan found off the west coast of Sweden. Although gametes had been reported, no larvae had been found. Almost nothing was known concerning its ecology.

More than 50 years later, it is now likely that *Xenoturbella* forms a new phylum, the ‘Xenacoelomorpha,’ together with the Acoelomorpha [5], but whether this new phylum belongs to the deuterostomes [5] or represents an early branching bilaterian [4] remains undetermined. Swimming stages during development have been reported [29], but its gastrulation has not been observed. Asexual reproduction is unlikely. It probably feeds on bivalves [22, 34, 35, 37], but the exact mode of feeding remains a mystery. Almost nothing is known concerning the functions of its two endosymbiotic bacteria [39, 40]. Thus, although a great deal of new scientific data has been reported for *Xenoturbella*, with the observation of developmental stages being a milestone, many questions have yet to be answered.

The main reason that so much important information remains unknown or unstudied is the difficulty in obtaining live specimens. The only population currently available for research is found from the Gullmarsfjord on the west coast of Sweden. To make matters more difficult, the population there is known to fluctuate from year to year, and there have been periods when no animals were collected for several years. Concerning developmental studies, obtaining mature adults at or prior to the winter breeding season is essential. However, during especially severe winters, Gullmarsfjord freezes and *Xenoturbella* collection cannot be performed for two to three months. One way to overcome this difficulty is to search for other *Xenoturbella* populations available for research. Gullmarsfjord is known for its biodiversity of animals that are usually found in much deeper seas, and this is the case more generally for other fjords [41–45]. Organisms that are typically found at depths of several hundred meters can be collected at around 100 meters in these fjords. If this is the case for *Xenoturbella*, the animal may inhabit the deep sea floor in other locations. Although scarce, there have been reports from the Norwegian coast, the North Sea, and the Adriatic Sea [33], and future deep-sea expeditions may discover new *Xenoturbella* species.

Why then do we have to study *Xenoturbella*? *Xenoturbella* is a zoological riddle that has puzzled biologists for over 60 years. Many mysteries, such as its phylogeny, complete development, and ecology remain. Studies on these subjects, together with research on its genome, gene regulatory networks, and neurology may uncover important clues for elucidating metazoan evolution. If it is in fact a deuterostome, it will be just the fourth extant deuterostome phylum, together with the hemichordates, echinoderms, and the chordates to which we humans belong, and studies on the animal may be useful for reconstructing the deuterostome ancestor.
